# Geriatric Comanagement Reduces Hospital-Acquired Geriatric Syndromes in Older Vascular Surgery Inpatients

**DOI:** 10.1093/geroni/igab046.1261

**Published:** 2021-12-17

**Authors:** Janani Thillainadesan, Sarah Aitken, Sue Monaro, John Cullen, Richard Kerdic, Sarah Hilmer, Vasi Naganathan

**Affiliations:** 1 Concord Hospital, NSW, New South Wales, Australia; 2 University of Sydney, Concord, New South Wales, Australia; 3 Concord Hospital, Concord, New South Wales, Australia; 4 The University of Sydney, St Leonards, New South Wales, Australia

## Abstract

Aims Based on our meta-analysis, surveys and qualitative studies of geriatricians in Australia and New Zealand, we designed and implemented a novel inpatient model to co-manage older vascular surgical inpatients at a tertiary academic hospital in Sydney. This model, called Geriatrics co-management of older vascular surgery patients (Gerico-V), embedded a geriatrician into the vascular surgery unit who introduced a range of interventions targeting older people. Here we evaluated this model of care. Methods We undertook a prospective before-and-after study of consecutive patients aged ≥65 years admitted under vascular surgery. One hundred and fifty-two GeriCO-V patients were compared with 150 patients in the pre- GeriCO-V group. The primary outcomes were hospital-acquired geriatric syndromes, delirium, and length of stay. Results The GeriCO-V group had more frail (43% vs 30%), urgently admitted (47% vs 37%), and non-operative patients (34% vs 22%). These differences were attributed to COVID-19. GeriCO-V patients had fewer hospital-acquired geriatric syndromes (49% vs 65%; P =.005) and incident delirium (3% vs 10%; P = .02), in unadjusted and adjusted analyses. Cardiac (5% vs 20%; P <.001) and infective complications (3% vs 8%]; P = .04) were fewer in the GeriCO-V group. LOS was unchanged. Frail patients in the GeriCO-V group experienced significantly less geriatric syndromes and delirium. Conclusions The Gerico-V model of care led to reductions in hospital-acquired geriatric syndromes, delirium, and cardiac and infective complications. These benefits were seen in frail patients. The intervention requires close collaboration between surgeons and geriatricians, and may be translated to other surgical specialties.

